# Conditionally unbiased estimation in phase II/III clinical trials with early stopping for futility

**DOI:** 10.1002/sim.5757

**Published:** 2013-02-15

**Authors:** Peter K Kimani, Susan Todd, Nigel Stallard

**Affiliations:** a Warwick Medical School, The University of WarwickCoventry, CV4 7AL, U.K.; b Department of Mathematics and Statistics, The University of ReadingReading, RG6 6AX, U.K.

**Keywords:** adaptive seamless designs, phase II/III clinical trials, estimation

## Abstract

Seamless phase II/III clinical trials combine traditional phases II and III into a single trial that is conducted in two stages, with stage 1 used to answer phase II objectives such as treatment selection and stage 2 used for the confirmatory analysis, which is a phase III objective. Although seamless phase II/III clinical trials are efficient because the confirmatory analysis includes phase II data from stage 1, inference can pose statistical challenges. In this paper, we consider point estimation following seamless phase II/III clinical trials in which stage 1 is used to select the most effective experimental treatment and to decide if, compared with a control, the trial should stop at stage 1 for futility. If the trial is not stopped, then the phase III confirmatory part of the trial involves evaluation of the selected most effective experimental treatment and the control. We have developed two new estimators for the treatment difference between these two treatments with the aim of reducing bias conditional on the treatment selection made and on the fact that the trial continues to stage 2. We have demonstrated the properties of these estimators using simulations. Copyright © 2013 John Wiley & Sons, Ltd.

## 1. Introduction

Modern innovations in clinical trial design have led to the availability of new approaches referred to as adaptive seamless designs (ASDs). Using an ASD, a clinical trial is conducted in 2 or more stages with interim analyses performed before the final stage to make adaptations. In this paper, we will consider two-stage ASDs where several doses or formulations of a drug, or several different treatments, are simultaneously compared with a standard/control with the poorly performing treatments dropped at stage 1 on the basis of interim analysis results. Such a trial is often termed a seamless phase II/III clinical trial. Unlike the traditional approach in which promising treatments are selected in a phase II trial separate to a confirmatory phase III trial, seamless phase II/III clinical trials combine aspects of both phases into a single trial with two or more stages. At the end of stage 1 of a two-stage seamless phase II/III clinical trial, an interim analysis is conducted to select the most promising treatment so that stage 1 resembles a phase II trial. The selected treatment together with the control treatment continues to stage 2 after which a confirmatory analysis is performed so that stage 2 resembles a fixed-sample-size phase III trial. The confirmatory analysis includes data from stages 1 and 2.

An ASD such as that described earlier poses a number of statistical challenges in both hypothesis testing and estimation of treatment effects because at the end of the trial, they use the data used in treatment selection to make inferences. An appropriate hypothesis testing method must be used to ensure that the overall type I error rate of the trial is not inflated. The evidence from the two stages can be combined using sufficient statistics from the accumulated data after each of the two stages or using the *p*-values from the two stages. Several authors 1–3 have proposed ASDs for which hypothesis testing is based on the sufficient statistics for the selected treatment effect, where the selected treatment is that which is seen to be most effective in the trial. If the selected treatment is not the most effective but testing is performed as if the most effective treatment has been selected using the preceding methods, the test is conservative 4. Hypothesis testing following an ASD can be carried out by combining the *p*-values from stages 1 and 2 5, 6. This method of testing is very flexible with regard to the choice of the selection rule. The flexibility of this testing has been exploited to propose ASDs that use Bayesian techniques to make the selection but use frequentist methods for hypothesis testing 7–9. A third technique of testing hypotheses after an adaptive trial is by using the conditional error principle 10 as in the adaptive Dunnett test 11.

The focus in this paper is estimation following an ASD. Estimation in this context is challenging because experimental treatments are retained in the trial precisely because they appear to be the most promising. Data suggesting that one treatment is superior may arise by chance even if the treatment is not truly superior to the other experimental treatments. Although the estimates may be biased, the bias can be quantified only if the rule for selecting the most promising treatment is specified in advance 12. This is because bias is defined as an expectation and expectations are taken over all possible outcomes, requiring specification of the selection rule used. The most promising treatment may be chosen on the basis of effectiveness and other factors such as safety. In this paper, we will focus on selection where the most promising treatment is that which has the highest apparent effectiveness at the end of stage 1. For such a selection, the effectiveness of treatments chosen to remain in the clinical trial is likely to be overestimated.

Regulatory guidance 13, 14 indicates that the bias of estimates obtained following an ASD should be considered. Cohen and Sackrowitz 15 and Shen 16 have proposed methods for estimating the mean of the selected treatment. The Cohen and Sackrowitz estimator is unbiased, whereas the Shen estimator reduces the bias relative to the naive estimator that ignores selecting the most effective treatment to continue to stage 2 based on the observed stage 1 data. Stallard and Todd 17 have proposed a method for estimating the mean of the selected treatment and also the means of the treatments that are dropped at stage 1. Cohen and Sackrowitz 15 and Shen 16 assume that the trial always continues to stage 2, whereas Stallard and Todd 17 assume that the trial may stop either for futility (when none of the experimental treatments are sufficiently effective on the basis of stage 1 data) or for efficacy. In this paper, we extend these methods to the setting where the trial can stop at stage 1 for futility, but not efficacy. This setting is common in practice. We will derive new estimators for the treatment difference for the selected treatment when estimation is unbiased conditional on continuing to stage 2. This differs from the Stallard and Todd estimator because the Stallard and Todd estimator is derived to be approximately unbiased conditional on the selected treatment whereas the estimators we will derive in this paper are obtained conditional on the selected treatment and the fact that the trial continues to stage 2. The two new conditional estimators (the word conditional is used to emphasize that estimation is unbiased conditional on continuing to stage 2) that we will derive extend the Cohen and Sackrowitz estimator and the Stallard and Todd estimator.

Like Koopmeiners *et al.* 18, we believe estimation unbiased conditional on continuing to stage 2 is of practical importance because when the trial cannot stop for efficacy at stage 1, it is reasonable to be interested in making a claim only when the trial continues to stage 2. Also, unconditionally unbiased estimators, that is estimators that do not condition on the stage at which the trial stops, may be conditionally biased 19. Because of this, in 19, the authors proposed to obtain estimators unbiased conditional on the stage at which the trial stops.

We organized the remainder of the paper as follows. In Section 2.1, we describe the setting of interest while giving the notation. In Section 2.2, we derive an estimator that extends the Cohen and Sackrowitz estimator, and in Section 2.3, we derive expressions used to obtain an estimator that uses the Stallard and Todd principle. In Section 3, we present a worked example. We compare the various estimators using a simulation study in Section 4. The paper ends with a discussion in Section 5.

## 2. Estimating treatment difference after an adaptive seamless design

### 2.1. Setting and notation

As already mentioned, we will consider ASDs with two stages where stage 1 is used to select the most effective treatment and stage 2 is used for confirmatory analysis. Let *k* (*≽* 2) denote the number of experimental treatments available at stage 1 for comparison with the control treatment, with the experimental treatment showing the highest effectiveness based on stage 1 data selected to continue to stage 2 together with the control. Let the number of subjects allocated to each treatment at stage 1 be denoted by *n*_1_. We assume outcomes from treatment *i* (*i* = 0,1,…,*k*), with *i* = 0 corresponding to the control treatment, are normally distributed with mean *μ*_*i*_ and a known common variance *σ*^2^, so that the stage 1 sample mean for treatment *i* follows a normal distribution 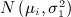, where 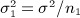. We denote the stage 1 sample mean for treatment *i* by *X*_*i*_ and the observed sample mean by *x*_*i*_. Let the selected treatment be denoted by *S* (*S* ∈ {1,…,*k*}), noting that *S* is a random variable, and the sample mean from the stage 2 data for treatment *i* (*i* = 0,*S*), with *i* = 0 corresponding to the control treatment, be denoted by *Y*_*i*_ with observed sample mean denoted by *y*_*i*_ so that 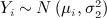, where 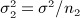, with *n*_2_ the number of subjects allocated to each treatment at stage 2. We suppose that the trial continues to stage 2 if *x*_*S*_ − *x*_0_ *≽ b*. We will refer to *b* as the futility boundary.

We define the selection time as the proportion *n*_1_ / (*n*_1_ + *n*_2_). This is the proportion of stage 1 data for the control and the selected treatment. We denote the selection time by *t* so that the sample mean from the two stages for the control treatment is given by *Z*_0,MLE_ = *tX*_0_ + (1 − *t*)*Y*_0_ and the sample mean for the selected treatment is given by *Z*_*S*,MLE_ = *tX*_*S*_ + (1 − *t*)*Y*_*S*_. After completion of the trial, the objective is to estimate the treatment difference *θ*_*S*_ = *μ*_*S*_ − *μ*_0_. We can base the inference on the naive maximum likelihood estimator (MLE) for the difference between the selected and control treatments given by

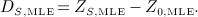
(1)

We will refer to this as the naive estimator. When there is no opportunity to stop at stage 1, the naive estimator is positively biased 12, 20,21. This is because the chosen experimental treatment is selected on the basis of having the maximum observed treatment difference compared with the control treatment.

In this paper, the objective is to seek estimators, which are unbiased conditional on continuing to stage 2, for the setting where a trial can stop at stage 1 for futility. For this setting, the naive MLE is also positively biased because of selecting the highest effective treatment and also requiring *x*_*S*_ − *x*_0_, the observed difference between the selected and control treatments at stage 1, to exceed the critical value *b*. If estimation is conditional on continuing to stage 2, *Y*_*S*_ and *Y*_0_ are respectively unbiased estimators for *μ*_*S*_ and *μ*_0_ so that the stage 2 sample difference

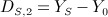
(2)is an unbiased estimator for *θ*_*S*_. However, this estimator, which we will henceforth refer to as the stage 2 estimator, is likely to be inefficient compared with estimators that use both stage 1 and 2 data. In Sections 2.2 and 2.3, we will derive two new estimators that use both stages 1 and 2 data.

### 2.2. A new unbiased estimator for the treatment difference

Cohen and Sackrowitz 15, although not considering the control treatment, derived a uniformly minimum variance unbiased estimator (UMVUE) for *μ*_*S*_ when the trial always continues to stage 2. When the trial always continues to stage 2, the bias of the naive estimator of *θ*_*S*_ arises from using *Z*_*S*,MLE_ as an estimator for *μ*_*S*_ 12. Thus, replacing *Z*_*S*,MLE_ with the Cohen and Sackrowitz UMVUE for *μ*_*S*_ in Equation (1) gives an unbiased estimator for *θ*_*S*_ in the case where the trial always continues to stage 2. In this paper, we are interested in a setting where a trial can stop for futility and estimation is conditional on continuing to stage 2. For this setting, *Z*_0,MLE_ is biased for *μ*_0_ and also the estimator for *μ*_*S*_ derived by Cohen and Sackrowitz is no longer unbiased because it does not condition on continuing to stage 2. In the rest of this section, we will derive the UMVUE for *μ*_*S*_ and the UMVUE for *μ*_0_ when estimation is conditional on continuing to stage 2, and hence an unbiased estimator for *θ*_*S*_.

The UMVUEs are based on the Rao–Blackwell theorem (for example 22). If estimation is conditional on continuing to stage 2, *Y*_*S*_ is an unbiased estimator of *μ*_*S*_. In the Rao–Blackwell theorem, a new estimator defined as the expected value of *Y*_*S*_ given a sufficient statistic for *μ*_*S*_ is the UMVUE for *μ*_*S*_. Similarly, *Y*_0_ is an unbiased estimator of *μ*_0_ so that the expected value of *Y*_0_ given a sufficient statistic for *μ*_0_ is the UMVUE for *μ*_0_. Let *X*_(1)_ > *X*_(2)_ > … > *X*_(*k*)_ be the order statistics of stage 1 sample means so that *X*_*S*_ = *X*_(1)_. For the selected treatment *S*, we show in Appendix A that the UMVUE for *μ*_*S*_, which we denote by *Z*_*S*,CHN_ with the notation chosen such that it reflects the fact that the estimator extends the Cohen and Sackrowitz UMVUE for *μ*_*S*_, is given by

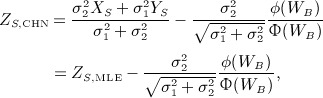
(3)
where *ϕ*(⋅) and Φ(⋅) respectively denote the density and distribution functions of a standard normal,

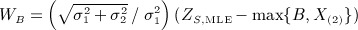
(4)
and *B* = *X*_0_ + *b*. For the control treatment, we show in Appendix B that the UMVUE for *μ*_0_, which we denote by *Z*_0,CHN_, is given by

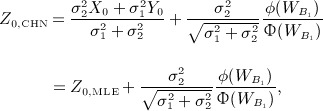
(5)
where

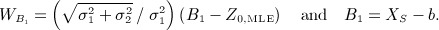


Because *Z*_*S*,CHN_ and *Z*_0,CHN_ are unbiased estimators for *μ*_*S*_ and *μ*_0_, then

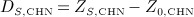
(6)
is an unbiased estimator for *θ*_*S*_. We will refer to this estimator as the (new) unbiased estimator. If we set the futility boundary *b* = − ∞ so that *B* = − ∞, *B*_1_ = ∞ and max{*B*,*X*_(2)_} = *X*_(2)_, then Equation (5) simplifies to

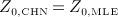

and Equation (4) simplifies to

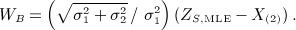
(7)

The simplification of *Z*_0,CHN_ to *Z*_0,MLE_ supports the finding in 12 that when there is no opportunity to stop at stage 1, the bias when the naive estimator is used as an estimator of the treatment difference is only contributed to by using *Z*_*S*,MLE_ as an estimator for the selected treatment in Equation (1). If further we have *σ*^2^ = 1 and *n*_1_ = *n*_2_ = 1 so that 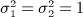, formulae (3) and (7) reduce to the formulae given by Cohen and Sackrowitz.

Koopmeiners *et al.* 18 considered the setting with *k* = 1. Note that for a trial with no control arm, the UMVUE for this setting is given by Equation (3), but with *W*_*B*_ expressed as
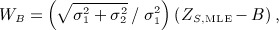
(8)where *B* is the futility boundary and *Z*_*S*,MLE_ is the sample mean for the experimental treatment. The same formula applies for the case with a control, replacing *Z*_*S*,MLE_ with *D*_*S*,MLE_, the sample difference between the experimental treatment and the control, and appropriately defining 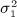 and 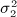 as the variances of stages 1 and 2 sample differences. Koopmeiners *et al.* also derived the UMVUE for the setting with *k* = 1. The formula given by Koopmeiners *et al.* has a typological error. Defining 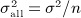, where *n* = *n*_1_ + *n*_1_, using our notation, they give the formula for UMVUE as
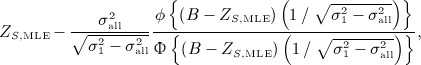
where *B* is the futility boundary, instead of
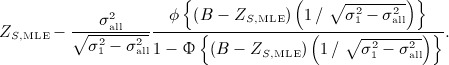


This formula can be shown to be equivalent to our formula. Thus, the Koopmeiners *et al.* estimator is a special case of our estimator.

### 2.3. A new bias-adjusted estimator for the treatment difference

Stallard and Todd 17 proposed a bias-adjusted estimator that involves estimating the bias of the naive estimator. A bias-adjusted estimate is then obtained by subtracting the estimate of the bias from the naive estimate. The bias-adjusted estimator is obtained as follows. Let 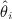 (*i* = 1,…,*k*) denote the naive maximum likelihood estimate for the treatment difference *θ*_*i*_ = *μ*_*i*_ − *μ*_0_. For *i* ≠ *S*, 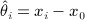, and for *i* = *S*, 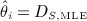. If the true vector ***θ*** = (*θ*_1_,…,*θ*_*k*_) ′ was known, the biases for the treatment differences of the naive maximum likelihood estimate 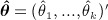 could be derived. Let the bias of 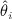 be denoted by *b*_*i*_(***θ***) and the vector (*b*_1_(***θ***),…,*b*_*k*_(***θ***)) ′ be denoted by ***b***(***θ***). Then, for example, the bias-adjusted estimator for *θ*_*S*_ could be given by *D*_*S*,MLE_ − *b*_*S*_(***θ***). However, the true mean vector ***θ*** is unknown. Following 23, Stallard and Todd proposed estimating the bias vector by iteratively solving 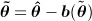, where 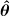 is the naive maximum likelihood estimate of ***θ***. The initial value 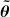 in the iteration procedure could be set to be 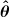. If the solution is achieved at iteration *r*, then the bias-adjusted estimator for *θ*_*S*_ is given by
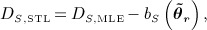
(9)where the notation is chosen to reflect the fact that this estimator is obtained using the principles described by Stallard and Todd.

Stallard and Todd derived the bias vector conditional on the selected treatment. Let stage 1 treatment differences *X*_*i*_ − *X*_0_ (*i* = 1,…,*k*) be denoted by *D*_*i*_ and the observed differences *x*_*i*_ − *x*_0_ by *d*_*i*_. One of the densities Stallard and Todd need while deriving the bias vector, which we also need in this paper, is the joint density of *S* = *i* and *d*_*i*_ given by
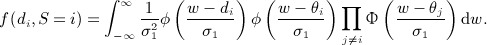
(10)

In the rest of this section, we will derive the bias vector when estimation is conditional on continuing to stage 2, where the trial continues to stage 2 if *d*_*S*_ *≽ b*. If the trial continues to stage 2, the expected value of the treatment difference for the selected treatment *i* is given by
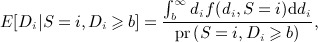
(11)where *f*(*d*_*i*_,*S* = *i*) is the density given by Equation (10). The numerator and the denominator in Equation (11) are simplified to expressions with single integrals in Appendix C.1. The expected value of the naive estimator given by Equation (1) can be expressed as *t*(*E*[*X*_*S*_ − *X*_0_] − *θ*_*S*_) + *θ*_*S*_ so that the bias of the treatment difference for the selected treatment *i*, given that the trial continues to stage 2, may be written as
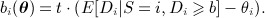
(12)

Conditional on the trial continuing to stage 2, the expected value of the treatment difference between a dropped treatment *i* ′ and the control treatment is expressed by
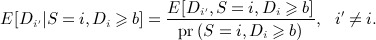


The expression for pr (*S* = *i*,*D*_*i*_ *≽ b*) is given earlier. The expression for 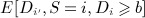 while using *D*_*i* ′_ directly involves multidimensional integrals that cannot be simplified to fewer integrals. To overcome this, we define a new variable *W*_0_ that has a normal distribution 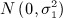 and its covariance with *D*_*i*_(*i* = 1,…,*k*), 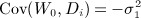. Then *W*_*i*_ = *D*_*i*_ + *W*_0_ is normally distributed with 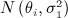 and Cov(*W*_*i*_,*W*_*j*_) = 0 for *i* ≠ *j* = 0,1,…,*k*. Note that
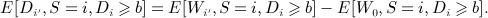


The expressions for *E*[*W*_*i* ′_,*S* = *i*,*D*_*i*_ *≽ b*] and *E*[*W*_0_,*S* = *i*,*D*_*i*_ *≽ b*] are simplified to single integrals in Appendix C.2. The bias of the treatment difference for a dropped treatment *i* ′ given that the trial continues to stage 2 may be written as
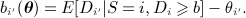
(13)

To obtain 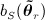 to substitute in Equation (9) and obtain a bias-adjusted estimate when estimation is conditional on continuing to stage 2, expressions (12) and (13) are used in the iteration procedure but with *θ*_*i*_ and 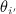 replaced by 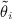 and 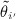, respectively. We will refer to this estimator for *θ*_*S*_ as the (new) bias-adjusted estimator. Koopmeiners *et al.* 18 derived a similar bias-adjusted estimator for the setting with *k* = 1 so that their bias-adjusted estimator is a special case of our bias-adjusted estimator.

## 3. Example

In this section, using the two new estimators described in Sections 2.2 and 2.3, we compute estimates for an example constructed from the case study described in 21. The case study is based on a comparison of three doses of an experimental drug for generalized anxiety disorder with a placebo. The primary endpoint is the change from baseline at 8 weeks of treatment in the total score on the Hamilton Rating Scale for Anxiety. The primary endpoint is taken to be normally distributed with a common standard deviation across the four treatment arms assumed to be 6 points. As in 21, we consider a two-stage ASD for the case study with *n*_1_ = *n*_2_ = 71 so that *t* = 0.5.

Suppose that the true treatment means are the stage 1 estimates from 21, which we give in Table 1 (column 1), and that the observed stage 1 means from an adaptive trial are as given in column 2. We suppose the trial continues to stage 2 if the highest effective experimental dose is at least as effective as the placebo, that is, the observed difference between the highest effective experimental dose and the placebo is at least 0. On the basis of the observed stage 1 data, dose 2 and placebo would be tested further in stage 2. We suppose the results from stage 2 are as given in column 3.

**Table I:** Data from a seamless phase II/III clinical trial.

		Treatment means				
		Observed		Treatment differences	
Treatment	True	Stage 1	Stage 2	Stage 1	Stage 2
Placebo	0	− 0.082	0.049	—	—
Dose 1	0.8	0.413	—	0.495	—
Dose 2	1.5	1.766	1.451	1.848	1.402
Dose 3	2.6	1.567	—	1.649	—

With the results in Table 1, *z*_0,MLE_ = (0.5 × − 0.082) + (0.5 × 0.049) = − 0.017 and *z*_2,MLE_ = (0.5 × 1.766) + (0.5 × 1.451) = 1.609 so that the naive maximum likelihood estimate for the difference between dose 2 and placebo is 1.626. The stage 2 estimate is 1.402 (column 5). For the new unbiased estimator, to compute *z*_2,CHN_, we note that *B* = − 0.082 + 0 = − 0.082 so that max{*B*,*x*_2_}is given by max{ − 0.082,1.567} = 1.567 and that 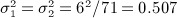 so that 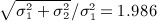 and 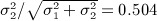. By substituting the appropriate values in Equations (3) and (4), *z*_2,CHN_ = 1.261. For *z*_0,CHN_, the only component we have not calculated is *B*_1_, which is given by 1.766 − 0. By substituting the appropriate values in Equation (5), *z*_0,CHN_ = − 0.017. Therefore, the unbiased estimate for the difference between dose 2 and placebo is 1.278.

For the new bias-adjusted estimator, we note that the naive maximum likelihood estimate for (*θ*_1_,*θ*_2_,*θ*_3_) is ( 0.495,1.626,1.649). The bias function for doses 1 and 3 is given by expression (13), and the bias function for dose 2 is given by expression (12). Using a program written in the R statistical package, we obtain the value of 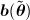 and hence 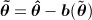 at each iteration. The iteration procedure stops at iteration *r* if the Euclidean distance between 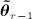 and 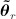 is less than or equal to 0.0005. The program is available at https://files.warwick.ac.uk/nstallard/browse/adaptive. We set 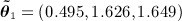. The iteration procedure stopped at iteration 15, and the bias-adjusted estimate for the difference between dose 2 and placebo is 1.135.

Thus, the naive, stage 2, unbiased, and bias-adjusted estimates for the difference between dose 2 and the placebo are 1.626, 1.402, 1.278, and 1.135, respectively. The estimates are different with the naive estimate, as expected, having the highest value. The unbiased and bias-adjusted estimates correct for the bias, and their values are below both the stage 1 and 2 differences. The unbiased and bias-adjusted estimates are closer to the stage 2 difference, which is an unbiased estimate of the treatment difference. We explore the properties of the four estimators in the next section.

## 4. Simulation study

### 4.1. Simulation study settings

In this section, we describe a simulation study that was used to assess the bias and the mean squared error (MSE) of the estimators described in Section 2. Following expressions (10), (11), and (12), the bias of the naive estimator depends on the number of experimental treatments *k*, the selection time *t*, the value of the futility boundary, and the true parameter values. Therefore, we will consider several scenarios in the simulation study. We will consider scenarios where *k* is between 2 and 5. We believe this encompasses the majority of practical scenarios with *k* > 1. We will also consider different true parameter values for the means. In all simulations, we will take the variance of the outcomes *σ*^2^ to be 1. Hence, we will only consider small differences in true treatment means corresponding to the small standardized effect sizes that we might anticipate in clinical trials.

We will assess three different values for the futility boundary. In most simulations that we will describe, we will take the treatment difference between the most effective treatment(s) and the control treatment to be 0.05. The first futility boundary value is 0, so because it is below the highest treatment difference(s), this boundary will be used to assess the bias when some of the experimental treatments are more effective than minimally required. The second futility boundary value is 0.05, so it will be used to assess the bias when the highest treatment difference is on the futility boundary. The third futility boundary value is 0.10, so it will be used to assess the bias when none of the experimental treatments are as effective as is minimally desired. We will also describe simulation results for some scenarios where the treatment difference between the most effective treatments(s) is 0.1, 0.2, and 0.5 while using the same futility boundary values (0, 0.05, and 0.1). These simulations will be used to assess the bias and MSE when most bias is contributed by the selection of the most effective treatment and not because of the futility boundary.

We perform simulations for 14 values of *t*, the selection time point, in the interval (0, 1). Because of the computations required, at each time point, we run 10,000 simulations that would continue to stage 2, that is, 10,000 simulations for which the simulated stage 1 treatment difference of the selected treatment is equal to or greater than the futility boundary value. For the treatment difference of the selected treatment *S*, in each simulation, we obtain the naive MLE *d*_*S*,MLE_ using Equation (1), the stage 2 estimate *d*_*S*,2_ using Equation (1), the unbiased estimate *d*_*S*,CHN_ using Equation (2), and the bias-adjusted estimate *d*_*S*,STL_ using Equation (9). We then calculate the differences (*d*_*S*,MLE_ − *θ*_*S*_), (*d*_*S*,2_ − *θ*_*S*_), (*d*_*S*,CHN_ − *θ*_*S*_), and (*d*_*S*,STL_ − *θ*_*S*_) and the respective squares (*d*_*S*,MLE_ − *θ*_*S*_)^2^, (*d*_*S*,2_ − *θ*_*S*_)^2^, (*d*_*S*,CHN_ − *θ*_*S*_)^2^, and (*d*_*S*,STL_ − *θ*_*S*_)^2^. Then at each selection time point, for each estimator, the mean bias is obtained by taking the average of its corresponding 10,000 differences and the MSE by taking the average of its corresponding 10,000 square differences.

We will present the bias and the 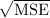 of the various estimators in units of the standard error (SE), the standard deviation for the estimator of the difference of a single experimental treatment–control comparison given by 
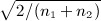
. This makes the results invariant to changes in the sample sizes.

### 4.2. Simulation results for *k* = 2 with *θ*_1_ = *θ*_2_

**Figure 1:**
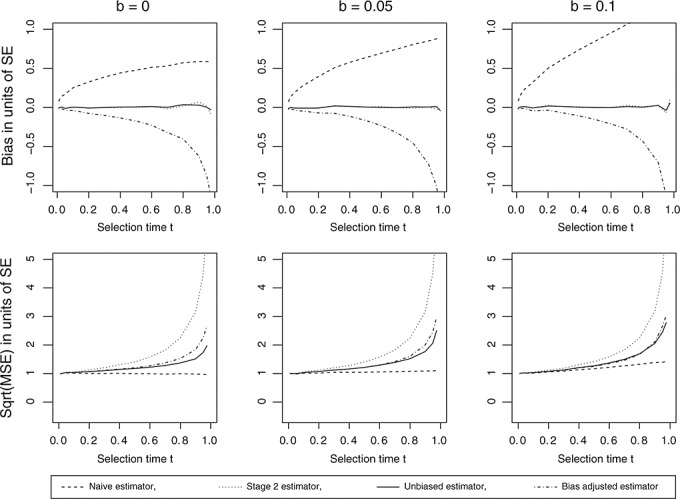
Bias (top row) and 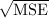 (bottom row) for various estimators. In all figures, *θ*_1_ = *θ*_2_ = 0.05. Dashed, dotted, continuous, and dash-dotted lines correspond to naive, stage 2, unbiased, and bias-adjusted estimators, respectively. The futility boundaries are given on top of each column. MSE, mean squared error; SE, standard error.

Figure 1 shows the bias and 4.2 when two experimental treatments and a control are included in stage 1 with *θ*_1_ = *θ*_2_ = 0.05. Columns 1, 2, and 3 correspond to futility boundary values 0, 0.05, and 0.1, respectively. The dashed, dotted, continuous, and dash-dotted lines correspond to the naive, stage 2, unbiased, and bias-adjusted estimators, respectively. The naive estimator is biased, and the bias increases with selection time but not linearly and also as the futility boundary value increases. The stage 2 estimator, as expected, is mean unbiased for all selection time points and all futility boundary values. Because of the theoretical derivation of the unbiased estimator, this is also mean unbiased for all scenarios. The bias-adjusted estimator overcorrects for bias, and the overcorrection increases with selection time but decreases as the value of the futility boundary increases. The naive estimator has the lowest MSE at all selection times for all scenarios. The stage 2 estimator has the highest MSE. In all scenarios, up to selection time 0.7, the unbiased estimator and the bias-adjusted estimator have approximately equal MSE. Tables giving more details of the results in Figure 1 and of additional simulations mentioned in the following are available from the authors.

We also assessed the characteristics of the four estimators when *θ*_1_ = *θ*_2_ = 0.1, *θ*_1_ = *θ*_2_ = 0.2, and *θ*_1_ = *θ*_2_ = 0.5 for futility boundary values *b* = 0, *b* = 0.05, and *b* = 0.1. We are able to clearly describe the findings from these scenarios without giving figures, and so we do not present figures for these scenarios. However, figures with these results and of additional simulations mentioned in the following, for which we have not presented figures, are available from the authors. For all the futility boundary values, the bias of the naive estimator decreases as the values of *θ*_1_ and *θ*_2_ increase. For *θ*_1_ = *θ*_5_ = 0.5, the biases of the naive estimator for *b* = 0, *b* = 0.05, and *b* = 0.1 are identical. This is because for this case, for the three futility boundary values, the stage 1 sample difference for experimental treatment 1 and the stage 1 sample difference for experimental treatment 2 beat the futility boundary values in most simulation runs so that the bias arises mostly because of the treatment selection and hence the similar bias of the naive estimator. As expected, the unbiased estimator and the stage 2 estimator are mean unbiased for all values of *t*, *b*, *θ*_1_, and *θ*_2_, whereas the bias-adjusted estimator is negatively biased, but the bias decreases as the values of *θ*_1_ and *θ*_2_ increase. For the three futility boundary values, as in the case where *θ*_1_ = *θ*_2_ = 0.05, for the cases where *θ*_1_ = *θ*_2_ = 0.1, *θ*_1_ = *θ*_2_ = 0.2, and *θ*_1_ = *θ*_2_ = 0.5, the naive estimator has the lowest MSE at all selection times, the stage 2 estimator has the highest MSE at all selection times, and up to selection time 0.7, the unbiased estimator and the bias-adjusted estimator have approximately equal MSE.

### 4.3. Simulation results for *k* = 2 with *θ*_1_ ≠ *θ*_2_

**Figure 2:**
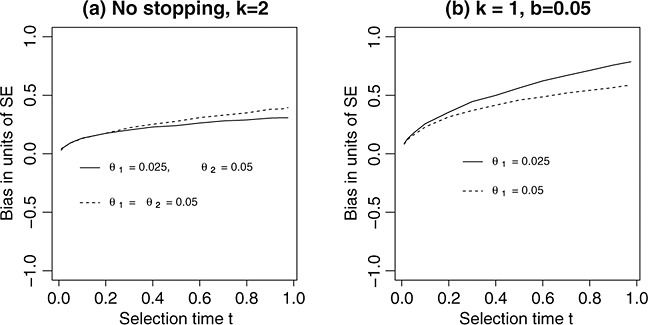
Panel (a) shows bias of the naive estimator when *k* = 2, and the trial always continues to stage 2 when *θ*_1_ = *θ*_2_ = 0.05 (dashed line) and when *θ*_1_ = 0.025 and *θ*_2_ = 0.05 (continuous line). Panel (b) shows bias of the naive estimator when *k* = 1 and futility boundary *b* = 0.05. The continuous and dashed lines correspond to *θ*_1_ = 0.025 and *θ*_1_ = 0.05, respectively.

To assess the bias and the MSE when *θ*_1_ ≠ *θ*_2_, we performed simulations with *θ*_1_ = 0.025 and *θ*_2_ = 0.05 for futility boundary values *b* = 0, *b* = 0.05, and *b* = 0.1. Most findings from these scenarios are similar to findings in Figure 1, and so we describe the findings of these scenarios without presenting a figure summarizing the results. As in the case when *θ*_1_ = *θ*_2_ = 0.05, the bias of the naive estimator increases with futility boundary value and the selection time when *θ*_1_ = 0.025 and *θ*_2_ = 0.05. As expected, the stage 2 and unbiased estimators are mean unbiased at all selection times and futility boundary values. In terms of bias, up to selection time 0.5, the bias-adjusted estimator performs almost as well as the stage 2 and unbiased estimators. The naive estimator has the lowest MSE whereas the stage 2 estimator has the highest MSE. The unbiased and bias-adjusted estimators have approximately equal MSE for all selection times and futility boundary values. For all the three boundaries considered, the bias of the naive estimator is slightly higher when *θ*_1_ = 0.025 and *θ*_2_ = 0.05 than when *θ*_1_ = *θ*_2_ = 0.05. The mean biases of the naive estimator for three values of *t* for the case where *θ*_1_ = *θ*_2_ = 0.05 and for the case where *θ*_1_ = 0.025 and *θ*_2_ = 0.05 are given in Table 2. This is unlike the setting in which the trial always continue to stage 2, where bias decreases as one of the experimental treatments becomes distinctly superior to the competing treatment 12. To assess what may be causing this difference, in Table 2, for both scenarios (*θ*_1_ = *θ*_2_ = 0.05 and *θ*_1_ = 0.025 and *θ*_2_ = 0.05), we present the probabilities of continuing to stage 2 (Pr[*d*_*S*_ *≽ b*]) and, conditional on continuing to stage 2, the simulated probabilities of selecting treatment 1 (Pr[*S* = 1 | *d*_*S*_ *≽ b*]), of selecting treatment 1 while treatment 2 also beats the futility boundary (Pr[*S*_1_,*d*_2_ *≽ b*]), and of selecting treatment 2 while treatment 1 also beats the futility boundary (Pr[*S*_2_,*d*_1_ *≽ b*]). As expected, for the case where *θ*_1_ = *θ*_2_ = 0.05, the simulated probabilities Pr(*S* = 1 | *d*_*S*_ *≽ b*) and Pr(*S* = 1,*d*_2_ *≽ b*) are respectively approximately equal to the simulated probabilities Pr(*S* = 2 | *d*_*S*_ *≽ b*) and Pr(*S* = 2,*d*_1_ *≽ b*). For both scenarios, from Pr(*d*_*S*_ *≽ b*), we observe that as the selection is made later in the trial, it is more likely that a right decision of whether to continue to stage 2 or not will be made. However, we note that for the case where *θ*_1_ = 0.025 and *θ*_2_ = 0.005, treatment 1 is still selected with relatively high probability (the minimum probability is 0.34 when *t* = 0.8 and boundary value *b* = 0.1). Also, when treatment 1 is selected to continue to stage 2, the treatment difference for treatment 2 is usually below the boundary ( Pr(*S* = 1,*d*_2_ *≽ b*) is small). We use Figure 2 to assess whether the instances where treatment 1 is selected are the ones that make bias higher when *θ*_1_ = 0.025 and *θ*_2_ = 0.05 than when *θ*_1_ = *θ*_2_ = 0.05. Figure 2(a) shows the bias of the naive estimator when *θ*_1_ = *θ*_2_ = 0.05 (dashed line) and when *θ*_1_ = 0.025 and *θ*_2_ = 0.05 (continuous line) in the case where the trial always continues to stage 2, and as expected, following 12, bias is higher when *θ*_1_ = *θ*_2_ = 0.05. The proof that for the case where the trial always continues to stage 2, the naive estimator is maximally biased when all experimental treatments are equally effective is given in 24. Figure 2(b) shows the bias of the naive estimator when *k* = 1 and the futility boundary value *b* = 0.05. The continuous and dashed lines correspond to *θ*_1_ = 0.025 and *θ*_1_ = 0.05, respectively. The bias is higher when *θ*_1_ = 0.025 than when *θ*_1_ = 0.05. Comparing Figure 2(a and b), we see that the futility boundary seems to contribute more to the bias. This may explain why in the case where there is a futility boundary and *k* = 2, the bias of the naive estimator is higher when *θ*_1_ = 0.025 and *θ*_2_ = 0.05 than when *θ*_1_ = *θ*_2_ = 0.05. Although the selected treatment may be the most promising because the treatment effects are distinct and hence reduce the selection bias, whenever the least effective treatment is selected, the bias is higher because we have a futility boundary.

**Table II:** Bias of the naive estimator and probabilities of various outcomes when two experimental treatments are tested in stage 2 for the case where *θ*_1_ = *θ*_2_ = 0.05 and the case where *θ*_1_ = 0.025 and *θ*_2_ = 0.05.

Characteristic	*t* = 0.2			*t* = 0.5			*t* = 0.8		
	*b* = 0	*b* = 0.05	*b* = 0.1	*b* = 0	*b* = 0.05	*b* = 0.1	*b* = 0	*b* = 0.05	*b* = 0.1
	*θ*_1_ = 0.05, *θ*_2_ = 0.05									
Mean bias	0.0239	0.0291	0.0336	0.0326	0.0453	0.0608	0.0386	0.0568	0.0807
Pr(*d*_*S*_ *≽ b*)	0.78	0.67	0.53	0.84	0.67	0.45	0.87	0.67	0.40
Pr(*S* = 1 | *d*_*S*_ *≽ b*)	0.49	0.50	0.50	0.50	0.49	0.50	0.50	0.50	0.51
Pr(*S* = 1,*d*_2_ *≽ b*)	0.29	0.25	0.21	0.33	0.25	0.19	0.34	0.25	0.16
Pr(*S* = 2,*d*_1_ *≽ b*)	0.30	0.24	0.21	0.32	0.26	0.18	0.34	0.25	0.16
	*θ*_1_ = 0.05, *θ*_2_ = 0.05									
Mean bias	0.0244	0.0293	0.0354	0.0360	0.0467	0.0631	0.0411	0.0615	0.0856
Pr(*d*_*S*_ *≽ b*)	0.76	0.64	0.50	0.80	0.62	0.41	0.83	0.61	0.34
Pr(*S* = 1 | *d*_*S*_ *≽ b*)	0.44	0.43	0.41	0.40	0.39	0.38	0.37	0.36	0.34
Pr(*S* = 1,*d*_2_ *≽ b*)	0.26	0.22	0.17	0.26	0.19	0.14	0.26	0.17	0.11
Pr(*S* = 2,*d*_1_ *≽ b*)	0.30	0.26	0.21	0.36	0.26	0.18	0.39	0.26	0.16

We also performed simulations when (*θ*_1_,*θ*_2_) = (0.075,0.1), (*θ*_1_,*θ*_2_) = (0.175,0.2), and (*θ*_1_,*θ*_2_) = (0.475,0.5) using the futility boundary values *b* = 0, *b* = 0.05, and *b* = 0.1. Note that for these parameter vectors, as for the case considered earlier where *θ*_1_ = 0.025 and *θ*_2_ = 0.05, *θ*_2_ − *θ*_1_ = 0.025. We describe the findings from these scenarios without giving the figures. For the three futility boundary values, the bias of the naive estimator decreases as the values of *θ*_1_ and *θ*_2_ increase. For (*θ*_1_,*θ*_2_) = (0.475,0.5), the biases of the naive estimator for *b* = 0, *b* = 0.05, and *b* = 0.1 are identical. This is because for this case, for the three futility boundary values, the stage 1 sample difference for experimental treatment 1 and the stage 1 sample difference for experimental treatment 2 beat the futility boundary values in most simulation runs so that the bias arises mostly because of the treatment selection and hence the similar bias of the naive estimator. For the three futility boundary values, compared with the case where (*θ*_1_,*θ*_2_) = (0.5,0.5), the biases of the naive estimator when (*θ*_1_,*θ*_2_) = (0.475,0.5) are lower. This is because, for futility boundary values 0, 0.05 and 0.1, stage 1 sample differences for treatments 1 and 2 beat the futility boundary values in most simulations for the cases where (*θ*_1_,*θ*_2_) = (0.5,0.5) and (*θ*_1_,*θ*_2_) = (0.475,0.5) so that most bias arises from treatment selection and selection bias is maximal when experimental treatments are equally effective 12, 24.

### 4.4. Simulation results for *k ≽*3

**Figure 3:**
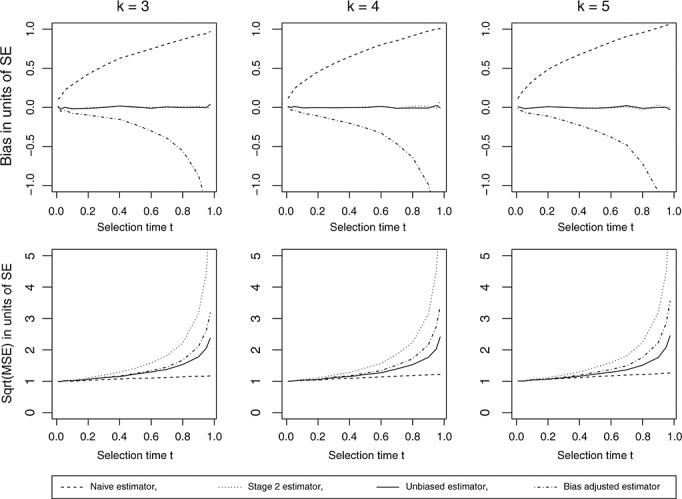
Bias (top row) and 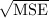 (bottom row) for various estimators when futility boundary *b* = 0.05. Dashed, dotted, continuous, and dash-dotted lines correspond to naive, stage 2, unbiased, and bias-adjusted estimators, respectively. The number of treatments is given on top of each column, and the treatment differences are all equal to 0.05.

When three or more experimental treatments are tested in stage 1, there are several possible configurations of the treatment differences, and this leads to several scenarios. Therefore, we will first describe general findings for such scenarios without presenting figures and then describe results of a few specific scenarios using a figure. On the basis of results that are not presented here, as in the case when two treatments are tested in stage 1, when three or more experimental treatments are tested in stage 1, the bias of the naive estimator increases with the futility boundary value, and estimation using the bias-adjusted estimator improves with higher futility boundary value whereas the stage 2 and unbiased estimators, as expected, provide unbiased estimators for all futility boundary values. Figure 3 shows results when treatment differences are all equal to 0.05 and the futility boundary is 0.05. Columns 1 to 3 give results when three, four, and five experimental treatments, respectively, are tested in stage 1. For the naive estimator, we observe that the bias increases slightly as the number of treatments increases. The stage 2 and unbiased estimators, as expected, are mean unbiased at all selection times and when three, four, or five experimental treatments are tested in stage 1. The bias-adjusted estimator again overcorrects for bias, and the overcorrection increases with selection time. Also, as the number of treatments increases, the overcorrection of the bias-adjusted estimator increases slightly. The naive estimator has the least MSE whereas the unbiased and bias-adjusted estimators have similar MSE for selection times up to 0.6. The stage 2 estimator has the highest MSE, and the difference between the MSE for the stage 2 estimator and the other estimators increases with selection time.

### 4.5. Summary of findings from the simulation study

From the simulation study, we have observed that the bias of the naive estimator increases with the selection time, the number of experimental treatments, and the futility boundary value. The treatment differences affect the bias of the naive estimator, but this also depends on the futility boundary value so that it is not possible to generalize the bias on the basis of treatment differences only. The stage 2 and unbiased estimators, as expected, provide mean unbiased estimates. The bias-adjusted estimator overcorrects for bias, but under some configurations of treatment differences, if selection is carried out up to selection time 0.4, it performs fairly well. For MSE, the unbiased and bias-adjusted estimators perform similarly up to time 0.6, whereas unsurprisingly, the stage 2 estimator performs worst. Regulation guidelines 13 suggest that methods for estimating treatment effect and confidence intervals with appropriate coverage should be provided as well as for controlling the prespecified type I error, whereas in 14, the importance of controlling the bias of the point estimate is emphasized. Hence, from the simulation findings and the importance of not overestimating treatment effect as described in 13, 14, we recommend the unbiased estimator.

## 5. Discussion

In drug development, the need to reduce the cost and time taken to test new treatments has led to the use of ASDs. ASDs combine several phases of a clinical development program into a single trial. However, compared with traditional testing strategies, ASDs pose additional challenges in statistical analysis. In this paper, we have considered point estimation following an ASD where, on the basis of observed data at stage 1, the experimental treatment that is superior to the competing experimental treatments at stage 1 continues to stage 2 together with the control. Cohen and Sackrowitz 15 and Shen 16 have considered this setting in the case where the trial always continues to stage 2 and proposed estimators for the treatment difference. Stallard and Todd 17 have also proposed an estimator that can be applied in this setting.

In this paper, we have considered the setting where the trial can stop for futility and estimation is unbiased conditional on continuing to stage 2. We have extended the Cohen and Sackrowitz method to construct an unbiased estimator for this setting. We have referred to this estimator as the (new) unbiased estimator. Carreras and Brannath 24 compared the Cohen and Sackrowitz estimator and the Stallard and Todd estimator when the trial always continues to stage 2. Their findings show that although the Cohen and Sackrowitz estimator is unbiased, it is not the best in terms of MSE. Thus, although the estimator we derive by extending the Cohen and Sackrowitz estimator to the setting where the trial can stop at stage 1 for futility is unbiased by construction, it is of interest to compare it with other estimators in terms of bias and MSE. Therefore, we have also developed a new bias-adjusted estimator that extends the Stallard and Todd estimator to our setting.

We also considered extending the Shen 16 estimator. The Shen estimator was proposed when the trial always continues to stage 2 and adjusts for bias by proposing a step function. When the trial always continues to stage 2, the step function depends on the absolute differences between the experimental treatment means and a tuning parameter. The best value for the tuning parameter depends on the unknown true values of the treatment means. With the possibility of early stopping, the bias depends not only on the absolute differences between the means of the experimental treatments but also on the values of observed differences between these and the mean of the control because of the futility boundary. This makes it challenging to propose a step function, and because we also know it will depend on a tuning parameter whose best value depends on the unknown true treatment means, we did not pursue this estimator further.

In terms of MSE, if treatment selection and the decision whether to continue to stage 2 are made at a selection time *t* < 0.6, the unbiased and bias-adjusted estimators perform similarly. The stage 2 estimator performs worst in terms of MSE, and the naive estimator (unadjusted for the possibility of stopping and for selection) performs the best. In terms of bias, the unbiased and stage 2 estimators are unbiased, and the naive estimator is positively biased whereas the bias-adjusted estimator is negatively biased. From this finding, we propose using the new unbiased estimator we have derived in this paper by extending the Cohen and Sackrowitz estimator 15 when a trial can stop for futility and estimation is performed conditional on continuing to stage 2. We emphasize that, although in the simulation study, we averaged over all simulations and the selected treatments, by derivation, the new unbiased estimator fulfills a stronger condition of unbiasedness in that it is unbiased with respect to each treatment whenever it is selected.

In this paper, we have considered point estimation following a two-stage adaptive seamless trial in which at stage 1, there is treatment selection and the possibility of early stopping for futility and estimation is conditional on the trial continuing to stage 2. As mentioned in Section 4.5, methods for interval estimation (confidence intervals) that adjust for the adaptation so that the right coverage is achieved are also important. There exist methods for constructing confidence intervals that can be used for the setting considered in this paper 17,20, 25. However, the confidence intervals following these methods are not based on the principle used to develop the estimators in this paper. For further research, we are considering confidence intervals based on the principle used to derive the unbiased estimator.

## Appendix A. Deriving the uniformly minimum variance unbiased estimator for *μ*_*S*_

For ease of notation in the derivation of UMVUEs for *μ*_*S*_ and *μ*_0_, without loss of generality, we let *X*_1_ > … > *X*_*k*_ so that *X*_(*i*)_ = *X*_*i*_ (*i* = 1,…,*k*) and *X*_*S*_ = *X*_1_ and *Y*_*S*_ = *Y*_1_. For the mean of the selected treatment, we are seeking the UMVUE for *μ*_1_. We will skip details of steps that are similar to steps given in 15 and 26. Denote by *Q*_*B*_(*X*) the event {*X*_0_, *X*_1_ > … > *X*_*k*_, *X*_1_ *≽ B*}, where *B* = *X*_0_ + *b*. We will first write and re-express the density of (*Y*_0_,*Y*_1_,*X*), where *X* = (*X*_0_,*X*_1_,…,*X*_*k*_) given *Q*_*B*_ in order to deduce the sufficient statistics for estimating *μ*_1_ that combine stage 1 and 2 means for treatment 1 into a single quantity. The density of (*Y*_0_,*Y*_1_,*X*) given *Q*_*B*_, denoted by *f*(*y*_1_,*y*_0_,*x* | *Q*_*B*_), is given by

where *σ*_1_ and *σ*_2_ are as defined in Section 2.1,  is the indicator for *Q*_*B*_(*x*), , and

The preceding density can be re-expressed as

where

Let ; then from the preceding density, (*X*_0_,*X*_2_,…,*X*_*k*_,*Y*_0_,*Z*_1_) is sufficient and complete for the problem of seeking an estimate for *μ*_1_ given *Q*_*B*_. Therefore, conditional on *Q*_*B*_, the UMVUE for *μ*_1_ is given by *E*[*Y*_1_ | *X*_0_,*X*_2_,…,*X*_*k*_,*Y*_0_,*Z*_1_,*Q*_*B*_]. We obtain the expression for this by deriving the density *f*(*y*_1_ | *x*_0_,*x*_2_,…,*x*_*k*_,*y*_0_,*z*_1_,*Q*_*B*_) and using it to get the expected value we are seeking.

Transforming the density *f*(*y*_1_,*y*_0_,*x* | *Q*_*B*_) into the density *f*(*x*,*y*_0_,*z*_1_ | *Q*_*B*_) gives

and transforming the density *f*(*y*_1_,*y*_0_,*x* | *Q*_*B*_) into the density *f*(*y*_1_,*x*_0_,*x*_2_,…,*x*_*k*_,*y*_0_,*z*_1_ | *Q*_*B*_) gives

The density *f*(*x*_0_,*x*_2_,…,*x*_*k*_,*y*_0_,*z*_1_ | *Q*_*B*_) is obtained from *f*(*x*,*y*_0_,*z*_1_ | *Q*_*B*_) by integrating out *x*_1_ as follows

where

We are seeking the density of (*Y*_1_ | *X*_0_,*X*_2_,…,*X*_*k*_,*Y*_0_,*Z*_1_,*Q*_*B*_), which is expressed as

Therefore, *E*[*Y*_1_ | *X*_0_,*X*_2_,…,*X*_*k*_,*Y*_0_,*Z*_1_,*Q*_*B*_] is equal to

Following 26, the numerator of the preceding expectation simplifies to

so that *E*[*Y*_1_ | *X*_0_,*X*_2_,…,*X*_*k*_,*Y*_0_,*Z*_1_,*Q*_*B*_] reduces to

and formula (3) gives the estimator when there is the possibility of stopping for futility and estimation is unbiased conditional on continuing to stage 2.

## Appendix B. Deriving the uniformly minimum variance unbiased estimator for *μ*_0_

Let . If we write and re-express the density *f*(*x*_0_,*x*_1_,…,*x*_*k*_,*y*_0_,*y*_1_) as in Appendix A, we can deduce that (*Z*_0_,*Y*_1_,*X*_1_,…,*X*_*k*_) is sufficient and complete for estimating *μ*_0_. Thus, we are seeking *E*[*Y*_0_ | *Z*_0_,*Y*_1_,*X*_1_,…,*X*_*k*_] so that if we denote by *X* the vector (*X*_1_,…,*X*_*k*_) ′, we need the density

Similar to derivation in Appendix A, it can be shown that the density *f*(*z*_0_,*y*_1_,*x*_0_,*x*) is given by

where

and  and *K*(*μ*) are as defined in Appendix A whereas the density *f*(*y*_0_,*z*_0_,*y*_1_,*x*) is given by

(14)

The density *f*(*z*_0_,*y*_1_,*x*) is obtained by integrating out *x*_0_ in the density *f*(*z*_0_,*y*_1_,*x*_0_,*x*). The range of *x*_0_ is − ∞ to *B*_1_ = (*x*_1_ − *b*) because the trial continues to stage 2 if (*x*_1_ − *x*_0_) *≽ b*. The density *f*(*z*_0_,*y*_1_,*x*) becomes

(15)

where

Using (14) and (15), we obtain

so that the expression for *E*[*Y*_0_ | *Z*_0_,*Y*_1_,*X*_1_,…,*X*_*k*_] is

(16)

The numerator can be re-expressed as

Similar to derivation in Appendix A, it can be shown that

so that the numerator in Equation (16)becomes

(17)

By substituting (17) in (16), *E*[*Y*_0_ | *Z*_0_,*Y*_1_,*X*_1_,…,*X*_*k*_] simplifies to

so that the UMVUE for *μ*_0_ is given by expression (5).

## Appendix C. Expressions for the expected values

### C.1 Probability of selecting a treatment and its expected value

The expression for the numerator in Equation (11) is given by

which by changing the order of integration becomes

As described in Appendices A and B, integration with respect to *d*_*i*_ is given by

so that the numerator in Equation (11) is given by

The pr(*S* = *i*,*D*_*i*_ *≽ b*), the denominator in Equation (11), is obtained by solving the integral , which can be simplified as follows

### C.2 Expected value for the dropped treatments

Without loss of generality, suppose treatment 1 is selected to continue to stage 2. Then for a dropped treatment *i* ′, the density of  when *d*_1_ *≽ b* is given by

which simplifies to

so that *E*[*W*_*i* ′_,*S* = 1,*D*_1_ *≽ b*] is

Changing the order of integration gives

From Appendices A and B, we can write the closed-form solution of the integration with respect to  so that the expression for *E*[*W*_*i* ′_,*S* = *i*,*D*_*i*_ *≽ b*] is

where

The density for *W*_0_ when treatment 1 is selected and *d*_1_ *≽ b* is given by

so that *E*[*W*_0_,*S* = 1,*D*_1_ *≽ b*] is given by

which by changing the order of integration becomes

When we use the results in earlier parts of the appendices, the solution for the integral with respect to *w*_0_ is − *σ*_1_*ϕ*{(*w*_1_ − *b*) / *σ*_1_}so that *E*[*W*_0_,*S* = 1,*D*_1_ *≽ b*] is given by
